# Striatal network dysfunction underlying speech impairment in early Parkinson’s disease

**DOI:** 10.1038/s41531-026-01509-2

**Published:** 2026-10-12

**Authors:** Caitlin Paige Rogers, Sandy Mournet, Cécile Gallea, Emma Biondetti, Graziella Mangone, Sara Sambin, Lydia Chougar, Nicolas Villain, Rahul Gaurav, Isabelle Arnulf, Marie-Odile Habert, Dijana Petrovska-Delacrétaz, Jean-Christophe Corvol, Habib Benali, Marie Vidailhet, Stéphane Lehéricy, Laetitia Jeancolas

**Affiliations:** 1 Institut du Cerveau – ICM, Inserm, CNRS, Sorbonne University, Paris, France; 2 Biological Sciences Department, Columbia University, New York, NY USA; 3 Department of Neurosciences, Imaging, and Clinical Sciences, Institute for Advanced Biomedical Technologies, ‘G. D’Annunzio’ University of Chieti-Pescara, Chieti, Italy; 4 AP-HP, Department of Neurology, Pitié-Salpêtrière Hospital, Paris, France; 5 AP-HP, Department of Neuroradiology, Pitié-Salpêtrière Hospital, Paris, France; 6 AP-HP, Sleep disorders unit, Pitié-Salpêtrière Hospital, Paris, France; 7 AP-HP, Department of Nuclear Medicine, Pitié-Salpêtrière Hospital, Paris, France; 8 CNRS, INSERM, Laboratoire d’Imagerie Biomédicale, LIB, Sorbonne University, Paris, France; 9 Laboratoire SAMOVAR, Télécom SudParis, Institut Polytechnique de Paris, Palaiseau, France; 10 Electrical & Computer Engineering Department, PERFORM Centre, Concordia University, Montreal, QC Canada

**Keywords:** Basal ganglia, Parkinson's disease, Functional magnetic resonance imaging

## Abstract

The pathophysiology underlying Parkinson’s disease (PD) speech impairments and its potential sex differences remain unclear. We investigated sex-specific alterations in speech and striato-cortical networks in 104 patients with early PD (68M/36F), and 44 healthy controls (23M/21F), using quantitative speech markers and multimodal neuroimaging. Speech features related to prosody, phonation, articulation, fluency, and rhythm, were extracted from voice recordings; striatal dopaminergic loss was quantified using DaT-SPECT, and functional connectivity between striatal and cortical language networks was analyzed with resting-state fMRI. Results showed that PD-related speech impairments were more pronounced in males. Functional connectivity was globally reduced in PD, but female patients exhibited higher caudate-cortical connectivity. In males, speech deficits were associated with both dopaminergic loss and connectivity disruption, whereas in females, only connectivity changes were involved. These findings revealed sex-specific pathophysiological mechanisms in early PD speech disorders, emphasizing the need for sex-stratified approaches in research and clinical practice.

## Introduction

Speech disorders, commonly described as hypokinetic dysarthria^1^, are among the first signs to appear in Parkinson’s disease (PD) and can manifest several years before a formal diagnosis is given^2,3^. They include prosody reduction (monopitch and monoloudness), phonation irregularities (pitch and intensity instability and hoarse voice), disruptions in articulation (vowel dedifferentiation and imprecise consonant articulation), abnormal pauses (jerky speech), and rhythmic impairments (difficulty maintaining a steady and imposed pace)^4,5^. Recently, many studies have characterized the voice and speech changes in the early stages of PD^6–12^. However, there is still a large knowledge gap in understanding the neural mechanisms underpinning such changes.

The pathophysiology of voice and speech impairment in PD, particularly the role of dopaminergic influence, is a matter of debate. Findings concerning the effects of dopaminergic treatment on Parkinsonian dysarthria are somewhat conflicting. Particularly, dopaminergic medication appears to have relatively beneficial effects on some speech performance in the early stages of the disease^13,14^. However, dysarthria seems to become unresponsive to dopaminergic treatment in more advanced stages^15^.

Previous studies have reported structural and functional changes in PD patients in the basal ganglia and several cortical regions that support normal speech production. Structurally, decreased cortical thickness of the precentral and fusiform cortices has been described in PD patients with hypokinetic dysarthria^16^, as well as bilateral posterior superior temporal gyrus gray matter loss compared to healthy controls (HCs)^17^. Functional activation studies during speech tasks have shown hypoactivation of the caudate nucleus, cerebellum, primary motor cortex, and pons, along with hyperactivation of the pre-supplementary motor area (pre-SMA), SMA, left dorsolateral prefrontal cortex, premotor cortex, inferior frontal gyrus, insula, and temporal gyrus^18–22^ in PD patients compared to HCs.

By analyzing functional magnetic resonance imaging (fMRI) data in a task-free context, researchers can make inferences about the intrinsic organization of functional brain networks that might otherwise be masked by the effects of task performance. Resting-state functional connectivity studies have reported reduced connectivity in PD compared to HC in speech networks involving the left thalamus, bilateral putamen, right rolandic operculum, and right superior temporal gyri^23^, as well as reduced connectivity in the speech prosody control network involving the orofacial sensorimotor cortex, the anterior cingulate, and caudate nucleus^24^. Wu et al.^25^ found lower resting-state functional connectivity in PD patients in areas involved in motor planning and coordination for speech, such as the SMA and left putamen, and higher connectivity in regions involved in motor execution and real-time adjustments during speech, like the left cerebellum and primary motor cortex. Others did not find any significant differences in resting-state functional connectivity between PD patients and HC^17^.

These studies have yielded various, sometimes contradictory, results and have rarely considered the influence of sex or the correlations with quantitative speech features. However, PD-related speech impairments are known to be sex-dependent, with more pronounced and detectable disruptions in males^5,7^. To address these limitations, we investigated whether these behavioral differences had any neurofunctional correlates. Our aim was to explore the neural mechanisms underlying PD-related speech impairments specific to each sex. To achieve this, we examined how the striatal dopaminergic system and its functional interaction with cortical speech and language networks are affected in PD and correlate with quantitative speech impairments. We used dopamine transporter single-photon emission computed tomography (DaT-SPECT) to assess striatal dopaminergic dysfunction and resting-state fMRI (rs-fMRI) to study functional connectivity. Our goal was to provide a deeper understanding of the sex-specific neural underpinnings of hypokinetic dysarthria in PD, which may inform the development of tailored therapeutic strategies.

## Results

### Demographic and clinical characteristics

A total of 148 right-handed participants, including 104 PD patients (68M, 36F) and 44 age-matched HC (23M, 21F), were analyzed (Table 1). No significant differences in age, years of education, MoCA score, or delays between voice recordings, DaT-SPECT, or MRI examinations were observed between patients and HCs. PD patients had significantly lower Mattis scores and higher MDS-UPDRS Part III scores in the OFF state. Within the HC group, males and females showed no differences in the clinical scores. However, in the patient group, males had a higher average MDS-UPDRS Part III score than females, as well as lower Mattis and MoCA scores. No significant difference in the disease duration, defined as time since formal diagnosis, was found between males and females.

**Table 1:** Demographic and clinical characteristics of the subjects

	PD			HC			Disease effect
	Male	Female	*P* value	Male	Female	*P* value	*P* value
No. of subjects	68	36		23	21		
Age (years)	63.6 ± 9.6	64.4 ± 9.1	0.68^A^	61.8 ± 10.7	61.0 ± 8.8	0.79^A^	0.14^C^
Disease duration (years)	2.5 ± 1.4	2.8 ± 1.6	0.45^A^	-	-		
Hoehn & Yahr	2.0 ± 0.1	2.0 ± 0.0	0.48^B^	-	-		
MDS-UPDRS III off	34.2 ± 7.1	29.7 ± 6.1	**0.001**^A^	4.6 ± 3.7	4.9 ± 3.5	0.81^A^	**<0.0001**^C^
MDS-UPDRS III on	28.4 ± 6.8	24.5 ± 6.0	**0.005**^A^	-	-		
MoCA	26.8 ± 2.2	27.7 ± 2.5	**0.02**^B^	28.0 ± 1.4	28.2 ± 2.0	0.32^B^	0.07^Ca^
Mattis	137.7 ± 4.5	140.1 ± 3.5	**0.002**^B^	139.6 ± 3.5	141.0 ± 2.0	0.24^B^	**0.03**^Ca^
Education level	6.3 ± 1.2	6.1 ± 1.4	0.61^B^	6.3 ± 1.2	6.3 ± 1.1	0.91^B^	0.76^D^
LEDD (mg)	434 ± 299	351 ± 181	0.19^B^	-	-		
Delay between voice recordings and DaT-SPECT (months)	−0.2 ± 5.0	−0.4 ± 5.1	0.87^A^	−0.1 ± 4.3	−0.3 ± 5.4	0.88^A^	0.89^C^
Delay between voice recordings and MRI (months)	−1.0 ± 6.3	0.0 ± 8.1	0.51^A^	−1.6 ± 4.2	−1.8 ± 6.9	0.91^A^	0.35^C^

Data are represented as means ± standard deviations. Bold values indicate statistical significance (p<0.05).

*HC* healthy control; *LEDD* Levodopa Equivalent Daily Dose; *MDS-UPDRS III* Movement Disorder Society revision of the Unified Parkinson’s Disease Rating Scale part III; *MoCA* Montreal Cognitive Assessment; *Off* off medication; *On* on medication; *PD* Parkinson’s disease.

A: Welch’s *t*-test; B: Mann–Whitney *U*-test; C: two-way ANOVA; D: binomial GLM on binarized score.

^a^Transformation *f*(*x*) = sqrt(Max − *x*) to have a normal residual distribution.

### Speech features

Group differences in speech features are presented in Table 2. PD patients overall had significantly more monotonous speech (lower *SD log F0*), longer pause durations, difficulty maintaining a steady rhythm (higher *RSD*), and a worse (higher) articulation score than controls (6.3 < *F* < 24.0; 10^−5^ < *p*_corr < 0.02), with medium to large effect sizes (0.04 < *η*^2^ < 0.14). No significant difference between PD patients and controls was found after false discovery rate (FDR) adjustment for SD intensity, absolute rhythm, and phonation. The main effect of sex was significant for *SD log F0* (higher in women) and median pause duration (shorter in women), with 10.0 < *F* < 17.8 and 0.0003 < *p*_corr < 0.007. Moreover, there was a significant interaction effect between disease and sex for the articulation score (greater PD effect in males than females; *F* = 17.57; *p*_corr = 0.0003).

**Table 2:** Group differences in voice recordings

Features	Males		Females		Sex effect	PD effect			Interaction effect		
	PD	HC	PD	HC	*p*	*p*	*η*^2^	CI_*η*^2^	*p*	*η*^2^	CI_*η*^2^
SD log_10_ F0 (x10^−3^)	57 ± 10	71 ± 11	71 ± 14	76 ± 13	**0.000**	**0.000**	0.13	[0.04, 0.23]	0.23	**-**	**-**
SD Int (dB)	5.4 ± 0.5	5.5 ± 0.4	5.3 ± 0.3	5.4 ± 0.4	0.18	0.33	-	-	0.94	-	-
Pause duration (s)	0.73 ± 0.28	0.57 ± 0.23	0.55 ± 0.18	0.49 ± 0.15	**0.007**	**0.02**	0.04	[0.00, 0.12]	0.47	**-**	**-**
RSD rhythm	0.12 ± 0.09	0.07 ± 0.05	0.14 ± 0.11	0.10 ± 0.06	0.18	**0.02**	0.04	[0.00, 0.12]	0.85	**-**	**-**
Absolute rhythm (s)	0.22 ± 0.17	0.21 ± 0.16	0.25 ± 0.16	0.20 ± 0.12	0.70	0.33	-	-	0.81	-	-
Phonation score	0.08 ± 0.98	0.02 ± 0.50	-0.10 ± 1.0	-0.32 ± 0.61	0.18	0.36	-	-	0.81	-	-
Articulation score	0.57 ± 0.14	0.39 ± 0.12	0.52 ± 0.03	0.50 ± 0.02	0.23	**0.000**	0.14	[0.05, 0.25]	**0.000**	0.11	[0.03, 0.21]

Means and standard deviations of speech features related to prosody, pauses, rhythm, phonation, and articulation were computed from all the tasks performed by PD and HC participants. Group differences were assessed using two-way analyses of variance with disease status and sex as factors and with an interaction term. *P* values were adjusted according to false discovery rate correction. Bold values indicate statistical significance (Corrected p < 0.05). Partial Eta-squared (*η*^2^) and its 95% confidence interval were computed for significant results.

*CI* 95% confidence interval, *F0* fundamental frequency, *HC* healthy control, *Int* Intensity, *PD* Parkinson’s disease, *RSD* relative standard deviation, *SD* standard deviation.

### Neuroimaging data

Group differences in DaT-SBR are presented in Table 3. There was a main effect of the disease with PD patients showing reduced SBR in both the left and right associative and sensorimotor territories of the putamen and the caudate nucleus (65.3 < *F*; 10^−39^ < *p*_corr < 10^−11^; 0.47 < *η*^2^ < 0.91) and a main effect of sex in all regions (4.51 < *F* < 21.3; 10^−4^ < *p*_corr < 0.04), with higher SBR in females. There was no significant Disease-Sex interaction effect.

**Table 3:** Group differences in DaT-SPECT specific binding ratios

Region	Side	PD		HC		Sex effect	PD effect		
		Male	Female	Male	Female	*p*	*p*	*η*^2^	CI_*η*^2^
**Putamen** Associative	L	2.74 ± 0.51	2.90 ± 0.63	5.85 ± 0.66	6.20 ± 0.63	0.03	0.000	0.87	[0.83, 0.90]
	R	2.72 ± 0.70	3.09 ± 0.87	5.77 ± 0.66	6.06 ± 0.68	0.03	0.000	0.80	[0.72, 0.85]
**Putamen** Sensorimotor	L	2.40 ± 0.39	2.52 ± 0.46	5.84 ± 0.78	6.14 ± 0.64	0.04	0.000	0.91	[0.88, 0.93]
	R	2.44 ± 0.55	2.78 ± 0.73	5.48 ± 0.57	6.10 ± 0.60	0.001	0.000	0.86	[0.81, 0.90]
**Caudate** Associative	L	3.29 ± 0.69	3.67 ± 0.93	4.90 ± 0.49	5.42 ± 0.72	0.001	0.000	0.55	[0.40, 0.65]
	R	3.51 ± 0.89	4.15 ± 1.07	5.25 ± 0.72	5.78 ± 0.62	0.001	0.000	0.47	[0.31, 0.59]
**Caudate** Sensorimotor	L	2.86 ± 0.69	3.44 ± 0.82	4.64 ± 0.64	5.30 ± 0.74	0.000	0.000	0.60	[0.46, 0.69]
	R	3.10 ± 0.78	3.77 ± 1.02	4.69 ± 0.52	5.48 ± 0.65	0.000	0.000	0.50	[0.35, 0.62]

Means and standard deviations of DaT-SPECT specific binding ratios computed in the putamen and the caudate nucleus. Group differences were assessed using two-way analyses of variance with disease status and sex as factors and with an interaction term. *P* values were adjusted according to false discovery rate correction. There were no significant interactions between disease and sex.

*CI* 95% confidence interval, *HC* healthy control, *L* left, *PD* Parkinson’s disease, *R* right.

**Fig. 1:**
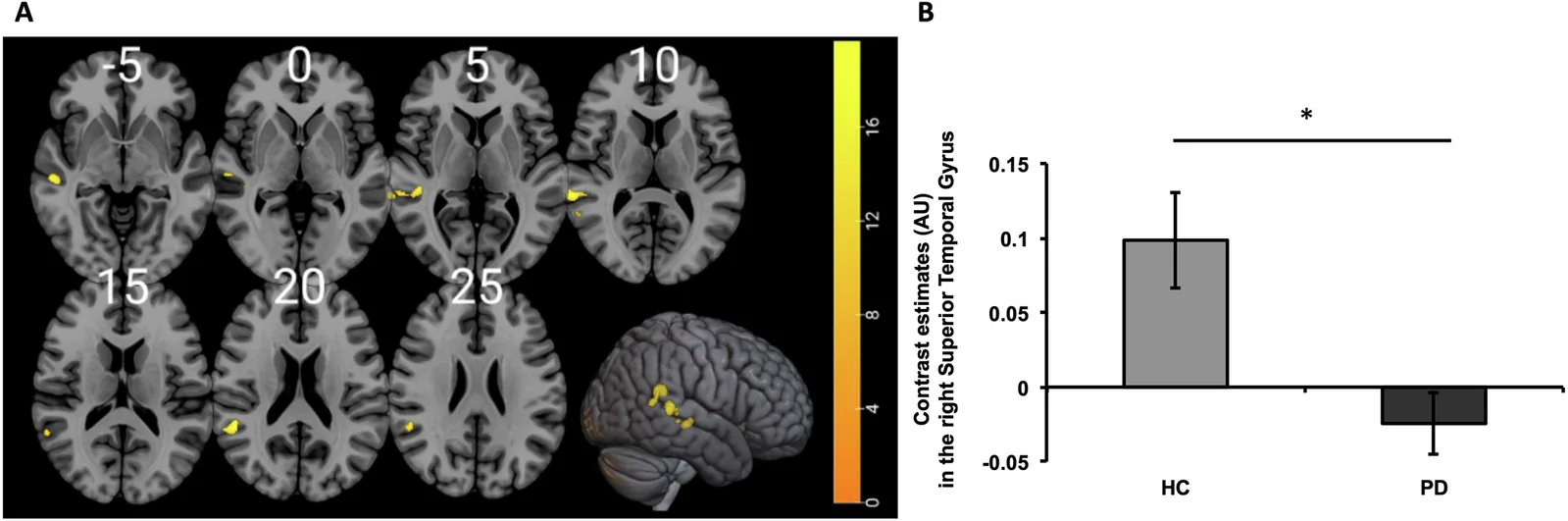
Disease effect on functional connectivity. **A** Areas corresponding to the significant disease main effect on striatal functional connectivity. Yellow: reduced functional connectivity in PD between the right and left associative caudate nuclei and the right superior temporal gyrus. The numbers correspond to the MNI *Z*-coordinates (axial views). **B** Plot of the PD and HC contrast estimates for the functional connectivity between the right associative caudate nucleus and the right superior temporal gyrus (the star indicates a significant difference at *p* < 0.05 with family-wise error correction at the cluster level).

**Fig. 2:**
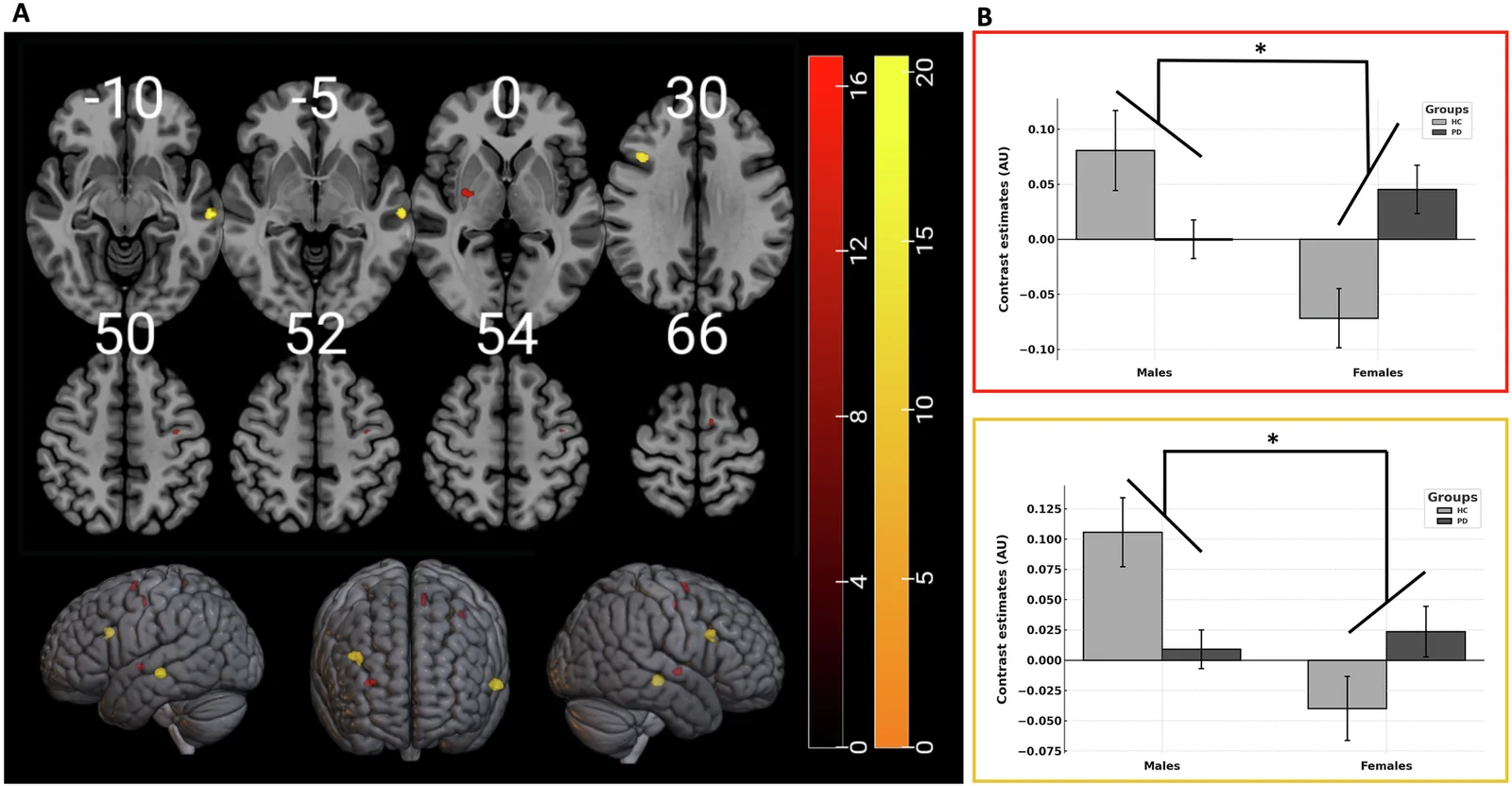
Disease-Sex interaction effect on functional connectivity*.* **A** Areas corresponding to a significant Disease-Sex interaction effect on striatal functional connectivity. Red: between the left sensorimotor caudate and the left precentral gyrus, the left supplementary motor area, and the right putamen. Yellow: between the right associative caudate and the left middle temporal gyrus and the pars triangularis of the right inferior frontal gyrus. The numbers correspond to the MNI *Z*-coordinates (axial views). **B** Plot of the contrast estimates for each subgroup for the functional connectivity between the left sensorimotor caudate nucleus and the left precentral gyrus (upper panel with red frame) and between the right associative caudate and the left middle temporal gyrus (lower panel with yellow frame). The stars indicate a significant interaction effect at *p* < 0.05 with family-wise error correction at the cluster level.

Group differences in functional connectivity are presented in Table 4. The ANOVA tests revealed a significant disease main effect (Fig. 1A) with decreased functional connectivity in PD observed between both the left and the right associative caudate nuclei and the right superior temporal gyrus (Fig. 1B). There was no significant effect of sex alone, but significant Disease-Sex interaction effects were observed (Fig. 2A). In two networks involving the caudate nucleus, we observed a PD effect in males (lower connectivity in PD than HC), which was reversed in females (Fig. 2B). The first network was composed of the left sensorimotor caudate, functionally connected to the left precentral gyrus, left supplementary motor area, and right putamen (red blobs in Fig. 2A). The second network was composed of the right associative caudate functionally connected to the left middle temporal gyrus and the pars triangularis of the right inferior frontal gyrus (yellow blobs in Fig. 2A). No significant group differences were observed in the functional connectivity with the putamen.

**Table 4:** Group differences in functional connectivity between striatal seed regions and language networks

Effect	Seed region	Target region	Side	BA	MNI coord (mm)			*P* cluster	Cluster size	*Z*_E_	*η*^2^	CI_*η*^2^
		(AAL3)			*X*	*Y*	*Z*					
**Sex**	-	-	-	-	-	-	-	-	-	-	-	-
**Disease**	**Caudate R** Associative	Temp Sup	R	22	64	−38	10	0.001	167	4.05	0.12	[0.04, 0.22]
	**Caudate L** Associative	Temp Sup	R	39	51	−45	20	0.000	232	4.20	0.13	[0.04, 0.23]
**Disease** **×** **Sex Interaction**	**Caudate R** Associative	Temp Mid	L	21	−63	−21	−9	0.001	148	4.38	0.13	[0.05, 0.24]
		Front Inf Tri	R	44	39	15	27	0.003	136	3.70	0.10	[0.03, 0.20]
	**Caudate L** Sensorimotor	Precentral	L	6	−36	−10	52	0.000	21	3.97	0.11	[0.03, 0.22]
		Putamen	R	Put	28	−9	−2	0.000	52	3.78	0.10	[0.03, 0.21]
		SMA	L	6	−10	−2	66	0.000	28	3.56	0.09	[0.02, 0.19]

Group differences were assessed using two-way analyses of variance with disease status and sex as factors and with an interaction term. Results were considered significant at the threshold of *p* < 0.001 uncorrected voxel-based level and of *p* < 0.05 with family-wise error correction at the cluster level. Only significant results are presented in the table. Partial Eta-squared coefficients (*η*^2^) and their 95% confidence intervals were computed at the peak voxel of each significant cluster.

*BA* Brodmann area, *CI* 95% confidence interval, *Front* frontal, *Inf* inferior, *L* left, *Mid* middle, *R* right, *Sup* superior, *SMA* supplementary motor area, *Temp* temporal, *Tri* pars triangularis.

It should be noted that the term “PD effect” or “disease effect” refers here to group differences between PD patients and HCs and does not imply a direct causal effect of the disease, as these differences may also reflect compensatory mechanisms or even medication effects.

### Correlations between speech features and neuroimaging

DaT-SBR only correlated with prosody, and only in males (Table 5). *SD log F0* positively correlated with DaT-SBR in the associative and sensorimotor territories of the putamen and caudate nucleus in the right hemisphere and the associative territory of the caudate nucleus in the left hemisphere (0.52 < *r* < 0.63; 0.01 < *p*_corr < 0.04). No significant correlation was found in females. No significant correlations were found between DaT-SBR and pause duration, RSD rhythm or articulation score.

**Table 5:** Correlation results between speech features and DaT-SPECT specific binding ratios in PD participants

Region	Territory	Side	SD log F0			Pause duration		RSD rhythm		Articulation	
			*r*	CI_*r*	*p*	*r*	*p*	*r*	*p*	*r*	*p*
**Males**											
Putamen	Associative	L	0.30	-	0.24	−0.27	0.24	−0.13	0.59	−0.15	0.55
		R	**0.53**	[0.35, 0.67]	**0.04**	−0.28	0.24	−0.16	0.53	−0.40	0.14
	Sensorimotor	L	0.29	-	0.24	−0.27	0.24	−0.08	0.68	−0.10	0.68
		R	**0.63**	[0.47, 0.75]	**0.01**	−0.28	0.24	−0.08	0.68	−0.33	0.24
Caudate	Associative	L	**0.52**	[0.34, 0.67]	**0.04**	−0.30	0.24	−0.27	0.24	−0.27	0.24
Nucleus		R	**0.56**	[0.39, 0.70]	**0.03**	−0.39	0.14	−0.26	0.26	−0.45	0.10
	Sensorimotor	L	0.41	-	0.14	−0.30	0.24	−0.17	0.50	−0.39	0.14
		R	**0.62**	[0.46, 0.74]	**0.01**	−0.33	0.24	−0.29	0.24	−0.46	0.10
**Females**											
Putamen	Associative	L	0.26	-	0.99	−0.35	0.99	−0.10	0.99	0.03	0.99
		R	0.02	-	0.99	0.08	0.99	0.03	0.99	0.04	0.99
	Sensorimotor	L	0.19	-	0.99	−0.11	0.99	0.22	0.99	0.07	0.99
		R	−0.10	-	0.99	0.16	0.99	0.20	0.99	0.13	0.99
Caudate	Associative	L	0.08	-	0.99	0.00	0.99	0.18	0.99	0.22	0.99
Nucleus		R	−0.01	-	0.99	0.23	0.99	0.17	0.99	0.14	0.99
	Sensorimotor	L	0.18	-	0.99	−0.05	0.99	0.23	0.99	0.23	0.99
		R	−0.09	-	0.99	0.33	0.99	0.13	0.99	0.31	0.99

Pearson’s *r* coefficients and *p* values resulting from the partial correlations, controlling for the age and the time delay between neuroimaging and voice recordings, between the discriminant speech features and the DaT-SPECT specific binding ratios, computed on the associative and sensorimotor territories of the putamen and caudate nucleus. All the correlations were computed in both hemispheres. *P* values were adjusted according to false discovery rate correction. Bold values indicate statistical significance (Corrected p < 0.05). 95% Confidence intervals of *r* coefficients were computed for significant results.

*CI* 95% confidence interval, *F0* fundamental frequency, *L* left, *PD* Parkinson’s disease, *R* right, *RSD* relative standard deviation, *SD* standard deviation.

All the significant correlation results between fMRI striatal connectivity and speech features are listed in Table 6 and described below.

**Table 6:** Correlation results between speech features and basal ganglia connectivity in PD participants

Sex	Seed region	Target region	Side	BA	MNI coord (mm)			*P* cluster	Cluster size	*Z*_E_	*r*	CI_*r*
		(AAL3)			*X*	*Y*	*Z*					
*Prosody (SD log F0)*												
Females	**Putamen L** Associative	Front Inf Oper	R	8	45	12	37.5	0.000	150	4.10	0.68	[0.45, 0.82]
		Front Sup Med	L	8	−7.5	46.5	48	0.037	60	3.95	0.66	[0.42, 0.81]
	**Putamen L** Sensorimotor	Front Mid 2	R	8	48	10.5	43.5	0.001	100	4.54	0.73	[0.52, 0.85]
		Thalamus VL	L	Thal	−13.5	−16.5	18	0.014	69	4.28	0.70	[0.48, 0.83]
	**Caudate L** Associative	Temp Sup	L	22	−58.5	−9	−3	0.000	293	3.97	−0.66	[−0.81, −0.42]
		Temp Mid	L	21	−60	−31.5	1.5	0.025	65	3.61	−0.61	[−0.78, −0.36]
	**Caudate L** Sensorimotor	Front Mid 2	R	6	34.5	3	52.5	0.043	60	3.64	−0.62	[−0.79, −0.36]
	**Caudate R** Associative	Temp Mid	L	21	−51	−36	−1.5	0.000	371	4.03	−0.67	[−0.82, −0.43]
		Front Inf Tri	R	9	54	24	28.5	0.001	96	3.61	−0.61	[−0.78, −0.36]
	**Caudate R** Sensorimotor	Front Mid 2	R	8	36	7.5	45	0.000	114	3.68	−0.62	[−0.79, −0.37]
Males	**Putamen L** Associative	Temp Mid	R	37	54	−52.5	3	0.000	218	3.98	−0.49	[−0.65, −0.28]
*Pause duration*												
Females	**Putamen L** Associative	Temp Mid	L	39	−55.5	−61.5	12	0.000	180	4.53	−0.73	[−0.85, −0.52]
		Temp Mid	L	22	−61.5	−10.5	−3	0.000	301	4.50	−0.72	[−0.85, −0.52]
		Front Inf Tri	L	44	−37.5	12	25.5	0.000	175	4.37	−0.71	[−0.84, −0.49]
	**Putamen R** Associative	Temp Sup	L	22	−57	−4.5	−4.5	0.001	100	3.81	−0.64	[−0.80, −0.39]
Males	**Caudate L** Associative	Front Sup Med	L	8	−7.5	39	49.5	0.002	142	3.88	−0.48	[−0.64, −0.27]
		Putamen	L	Put	−27	13.5	−9	0.044	87	4.02	0.49	[0.28, 0.65]
*RSD rhythm*												
Females	**Putamen R** Associative	Front Inf Oper	R	44	42	13.5	25.5	0.001	97	3.92	0.65	[0.41, 0.81]
*Articulation*												
Females	**Putamen L** Associative	Temp Mid	L	39	−54	−60	15	0.000	151	4.15	0.68	[0.46, 0.83]
	**Caudate R** Associative	Temp Mid	R	39	43.5	−55.5	12	0.042	60	3.74	0.63	[0.38, 0.80]
Males	**Caudate L** Associative	Temp Mid	L	37	−61.5	−63	9	0.046	86	3.66	−0.45	[−0.62, −0.24]

Multiple regression analysis between the speech features significantly impaired in PD and the functional connectivity scores between the basal ganglia seeds and the language-network area. Analyses were performed in PD patients, separately in men and women, and were controlled for age, education level and the time delay between neuroimaging and voice recordings. The speech features included (1) SD log F0, (2) the median pause duration, (3) RSD rhythm, and (4) the articulation score. Results were considered significant at the threshold of *p* < 0.001 uncorrected at the voxel-based level and of *p* < 0.05 with family-wise error correction at the cluster level. Only significant results are presented in the table. Correlation coefficients (*r*) and their 95% confidence intervals were computed at the peak voxel of each significant cluster.

*AAL3* Automated Anatomical Labeling 3, *BA* Brodmann area, *CI* 95% Confidence Interval, *Front* frontal, *F0* fundamental frequency, *Inf* inferior, *L* left, *Med* medial, *Mid* Middle, *MNI* Montreal Neurological Institute, *Oper* opercular, *PD* Parkinson’s disease, *R* right, *Sup* superior, *RSD* relative standard deviation, *Temp* temporal, *Tri* pars triangularis, *VL* ventrolateral.

*Prosody* (*SD log F0*) was positively correlated, in females, with connectivity between (1) the left sensorimotor putamen and the right middle frontal gyrus and the left ventrolateral thalamus; and (2) the left associative putamen and the left superior frontal gyrus (medial part) and the opercular part of the right inferior frontal gyrus. In addition, negative correlations were found with connectivity between (1) the left associative caudate and the left superior and middle temporal gyri; (2) the left sensorimotor caudate and the right middle frontal gyrus; (3) the right sensorimotor caudate and the right middle frontal gyrus; and (4) the right associative caudate and the triangular part of the right inferior frontal gyrus, and the left middle temporal gyrus. In males, a negative correlation was observed with connectivity between the left associative putamen and the right middle temporal gyrus.

Median *pause duration* was negatively correlated, in females, with connectivity between the left associative putamen and the triangular part of the inferior frontal gyrus, and the middle temporal gyrus in the left hemisphere, as well as between the right associative putamen and the left superior temporal gyrus. In males, a negative correlation was found with connectivity between the left associative caudate and the left superior frontal gyrus, and a positive one with connectivity between the left associative caudate and the left putamen.

RSD *rhythm* measures were positively correlated, in females, with connectivity between the right associative putamen and the opercular part of the right inferior frontal gyrus. No significant correlations were observed in males.

*Articulation* values were positively correlated, in females, with connectivity between the left associative putamen and the left middle temporal gyrus, as well as between the right associative caudate and the right middle temporal gyrus. In males, a negative correlation was observed with connectivity between the left associative caudate and the left middle temporal gyrus.

All correlation analyses presented here were controlled for age and education level when appropriate, thereby reducing additional sources of unexplained variability and spurious correlations. We also repeated the analyses with and without adjustment for the time delay between voice recording and imaging acquisition, and the main findings remained materially unchanged. Given its ability to account for an additional potential source of variability, the adjusted analysis was retained in the present article.

## Discussion

In this study, we explored the neural mechanisms underlying PD speech impairments. We particularly investigated how the striatal dopaminergic system and its functional interaction with the cortical language networks were affected in PD and how they correlated with speech dysfunction. PD patients exhibited significantly more monotonous speech, longer pause durations, difficulty maintaining a steady rhythm, and a worse articulation score than HCs. They also showed an overall decrease of striatal dopaminergic binding ratio and functional connectivity in the associative striatal networks. A Disease-Sex interaction was observed in the articulation score and in the functional connectivity between the caudate nucleus and several language regions.

The correlations between the neuroimaging data and speech features were highly sex-dependent in PD participants. In male patients, speech impairments were associated with striatal dopaminergic loss and disruptions in the functional connectivity between the associative striatum and fronto-temporal cortical areas. In female patients, speech deteriorations were only associated with functional connectivity changes, but involved more regions, including both the associative and sensorimotor striatal connectivity with fronto-temporal cortical areas.

Our findings reveal significant distinctions between PD patients and HCs across both behavioral and neurobiological measures, the implications of which are discussed in detail below.

*Speech impairments* in PD participants were primarily characterized by reduced intonation, assessed using *SD log F0*, and altered articulation, assessed using Mel-Frequency Cepstral Coefficients (MFCCs). PD patients also showed longer median pause durations, reflecting the impression of jerky speech^1^, and suggesting a cognitive component in addition to the motor component of the disorder. The RSD rhythm during the slow syllable repetitions was increased, showing the difficulty PD subjects had in keeping a steady rhythm. The absolute variation of the averaged pace compared to the imposed rhythm was not significantly impaired, but it was the case with a larger database^5^. Phonation and intensity fluctuations (*SD Int*) did not differ between PD and HCs, possibly due to the effect of the dopaminergic treatment^13^. The previously listed speech alterations were encountered in both males and females with PD, but overall were more pronounced in males, particularly the articulation.

*DaT-SBR* was found to be reduced in PD patients in the putamen and caudate nucleus, with a more pronounced decline in the sensorimotor putamen, consistent with the literature^26^. We also noted a sex effect in the entire striatum with higher SBR in females, in agreement with previous findings^27^.

*Functional connectivity* analyses revealed lower connectivity in PD patients between the associative caudate nucleus and associative cortical language network areas, particularly the right superior temporal gyrus, whose connectivity has previously been reported to be reduced in PD^23^. The stronger connectivity reduction observed in the associative striatum compared with the sensorimotor striatum might at first seem contradictory to the usual observation that hypoconnectivity is more pronounced in sensorimotor regions^28,29^. A possible explanation may be that BOLD images were acquired while patients were on medication, and it has been shown that the sensorimotor system is the main target of levodopa administration^30^. Moreover, when specifically considering the caudate nucleus, disruptions of its connectivity are usually observed with associative regions^31,32^. The PD-related decrease of functional connectivity was more pronounced in males, which is in line with a previous study^33^. Interestingly, connectivity was even increased in females with PD in two networks involving the caudate nucleus. An increased strength of striatal functional connectivity in females with PD has previously been reported in a smaller cohort^34^ and may reflect a form of functional reorganization, potentially compensatory. This interpretation, although hypothetical, may help explain the milder cognitive and motor impairments observed in women with PD in our cohort.

This study also provides new insight into how dopaminergic loss and functional connectivity changes contribute to speech impairment in PD patients, with distinct patterns observed between males and females. Based on the literature, we propose that reduced functional connectivity in the striato-cortical network may be considered a direct effect of the pathology and could contribute to speech impairments^35,36^. Conversely, high levels of functional connectivity may reflect a compensatory mechanism^37^ that, if efficient, would lead to milder speech disruptions, and if maladaptive or insufficient, would be associated with more severe speech impairments. Based on these hypotheses, for prosody, as SD log F0 was reduced in PD, a positive correlation (high connectivity associated with intact prosody) could indicate a direct effect of the disease or a successful compensation, while a negative correlation (high connectivity associated with impaired prosody) may suggest a maladaptive or insufficient compensatory mechanism. For pauses, rhythm, and articulation, these features increased with disease progression, so the direction of the correlations would convey the opposite meaning.

The *prosody* reduction (lower *SD log F0*) in PD participants’ speech likely results from decreased laryngeal movements caused by excessive rigidity and bradykinesia^13^. This diminished pitch variability was found in both males and females with PD; however, the neural mechanisms underlying prosody impairments differed by sex. Prosody impairments in males with PD were associated with dopaminergic loss in the putamen and caudate nucleus. This finding is in line with previous observations in PD patients reporting a role of the basal ganglia in prosody production and speech melody modulation^22,38^, and of dopaminergic depletion in impaired intonation^39–41^. Moreover, the correlation with striatal SBR was more significant in the right hemisphere, in agreement with the role of this hemisphere in prosody reported previously^42^, and even more pronounced in the sensorimotor territories, which underlie the motor component of prosody reduction. The fact that male but not female patients demonstrated a correlation between monopitch and dopaminergic loss in the striatum supports the results of a previous study on de novo PD^43^. As for females with PD, we showed that their prosody reduction was primarily associated with striatal functional connectivity disruptions. They showed correlations between the reduction of prosody and the decrease of connectivity between the left (sensorimotor and associative) putamen and the bilateral frontal cortex, as well as the left ventrolateral thalamus. This provides a quantitative and objective confirmation of the correlations observed previously between perceptually assessed speech impairments (speech items of UPDRS) and a decreased functional connectivity between the putamen and both the thalamus and the ventral premotor area^23^. In addition, we found several negative correlations between prosody and caudate-fronto-temporal functional connectivity, possibly hinting at a maladaptive or insufficient compensatory mechanism.

The lengthening of median *pause duration* observed in PD participants’ speech is the result of both a reduction in short pauses and an increase in long pauses. The reduced number of short pauses is caused by an incomplete closure of the vocal tract during stop consonants^6^, due to bradykinesia and rigidity, and is also related to an oral festination, which is an axial symptom^44^. The increased number of long pauses suggests the involvement of a cognitive component (e.g., syntax processing, speech planning, word selection, and memory), in addition to the motor dysfunction. The lengthening of median pause durations was associated in our study with a decrease in functional connectivity within associative striato-cortical networks, further emphasizing the cognitive aspect. Notably, the correlation found in females with PD between left putamen–left superior temporal gyrus connectivity and pause duration disruptions echoes the relationship observed in several studies^23,36^ where this specific PD-related hypoconnectivity was linked to perceptually assessed speech impairments.

The increased RSD *rhythm* observed in PD during slow syllable repetitions showed the difficulty PD subjects had in keeping a steady rhythm. This rhythmic impairment may be related to the loss of motor automaticity commonly observed in PD patients^45,46^ and to the dysfunctional processing of auditory feedback^22^. The positive relationship we observed in PD women, between the rhythmic difficulties and the connectivity between the right putamen and the opercular part of the inferior frontal gyrus, is coherent with previous evidence showing that the basal ganglia, including the putamen, and their interactions with frontal regions, is critical for beat perception and internal generation of rhythm^47^.

PD *dysarthria* is commonly characterized by vowel dedifferentiation^48^, due to an amplitude reduction of tongue and lip movements, and imprecise consonant articulation, with a spirantization of stop consonants that are pronounced as fricatives due to incomplete oral closure^49^. These articulatory disruptions modify the spectral envelope, making them well captured by the MFCCs, on which our articulation score is based. We observed positive correlations in PD women with the connectivity between both the left putamen and right caudate with the middle temporal gyrus, a region which plays a significant role in phonological processing and lexical access, essential for retrieving and producing words during speech^50^. These positive correlations, based on our hypotheses, may reflect a maladaptive neural response to the dopaminergic degeneration characteristic of PD. The hyperconnectivity between these regions may lead to disrupted timing and coordination between motor planning and linguistic processing, exacerbating articulation difficulties. Similar patterns of maladaptive hyperconnectivity have been reported in PD, where increased connectivity within motor and language networks correlates with symptom severity^51^. These findings suggest that the brain’s attempt to compensate for neural degeneration may inadvertently contribute to worsening motor symptoms, including those affecting speech.

We found that the neural correlates of PD speech impairments in the striatal dopaminergic system were clearly *sex-dependent* and may explain, at least partly, why speech impairments were more pronounced in males. It has already been shown that the PD phenotype is sex-dependent, with a milder form and a delayed onset in females, which could be a consequence of a possible protective role of estrogen in PD^52^. According to previous studies, females with PD exhibit less pronounced brain atrophy and fewer network disruptions^33^. In the same vein, we showed that females with PD had a higher dopaminergic reserve than males and increased caudate-cortical functional connectivity, which may reflect a compensatory mechanism. Nevertheless, despite balancing the duration since formal diagnosis between sexes, an unaccounted disparity in the time since symptom onset may exist. Consequently, the assumption of an identical stage of pathology between groups may not be entirely warranted. Finally, the speech neural circuits have been reported to be different in men and women^53–57^ with stronger bilateral activity in females. These circuits may therefore be differently affected in PD, leading to different types or degrees of speech impairments. In our study, we observed that some speech impairments were associated with dopaminergic loss in males with PD but none in females. In females, the speech disruptions were more associated with various changes in striato-cortical functional connectivity, suggesting an intricate functional reorganization in the subcortical-cortical network^29,58^.

Interpreting the directionality of the observed correlations between functional connectivity and speech impairments remains a complex task. It is difficult to clearly determine whether the alterations in connectivity reflect a direct effect of PD pathology or represent compensatory mechanisms engaged by the brain to counteract neural degeneration. Additionally, given that patients were assessed under treatment in the “ON” state, dopaminergic medication may have influenced both neural connectivity and speech parameters. This represents a limitation, as medication effects cannot be fully disentangled from disease-related effects, thereby further complicating the interpretation of the findings. The brain may reorganize its networks in response to both disease progression and treatment effects, leading to adaptive or maladaptive changes in connectivity. Given this ambiguity, it may be more appropriate to interpret these alterations in terms of functional reorganization rather than attributing them solely to direct disease effects or compensatory mechanisms. This perspective acknowledges that the brain undergoes complex changes that cannot be unequivocally classified as beneficial or detrimental without further investigation. Overall, this complexity calls for cautious interpretation of our findings and highlights the need for longitudinal studies to better understand the causal relationships involved and to clarify whether observed connectivity changes are beneficial adaptations, treatment effects, or detrimental consequences of disease progression.

Additionally, our use of rs-fMRI to investigate neural correlates of speech impairments has limitations. Resting-state connectivity analyses provide robust insights into the brain’s intrinsic functional architecture, which are not confounded by task activity. The ROIs that compose the healthy speech networks were selected from task-based mapping studies and anatomical atlases for a comparison of resting-state connectivity between the healthy brain and PD. However, some connectivity alterations might only appear during active vocal tasks and may remain undetected at rest. Therefore, combining resting-state and task-based approaches may offer an even more comprehensive perspective on the functional reorganization associated with speech deficits.

To conclude, in this study, we explored the neural mechanisms underlying PD speech impairments. We particularly investigated the role of the striatum and its functional connectivity with the cortical language networks in PD-related speech dysfunctions. We found that the neural correlates of PD-related speech impairments were highly sex-dependent. In PD males, speech disturbances were associated with dopaminergic loss in the striatum and changes in the associative striato-cortical functional connectivity. In females with PD, speech disturbances were linked primarily to changes in associative and sensorimotor striato-cortical functional connectivity. These results highlight the importance of sex-specific neural mechanisms in PD-related speech disorders and suggest that therapeutic approaches should consider these differences to improve patient outcomes.

## Methods

### Participants

Subjects were prospectively enrolled as part of the ICEBERG longitudinal study (ClinicalTrials.gov Identifier: NCT02305147). This cohort includes HCs and patients with PD. Inclusion criteria were a clinical diagnosis of idiopathic PD according to the United Kingdom Parkinson’s Disease Society Brain Bank^59^ with less than 4 years of disease duration and minimal or no cognitive disturbances (i.e., a Mini-Mental State Examination score ≥26/30)^60^. HCs had to be free of any neurological disorders. Left-handed subjects and participants with a gap of over 1 year between voice recordings and neuroimaging were excluded from the analyses to minimize variability in hemispheric lateralization and ensure temporal alignment between behavioral and neurofunctional data. Participants with PD were pharmacologically treated and performed the audio recordings and neuroimaging acquisitions during the 6 h following their last dopaminergic medication intake, so under the active effect (at least partial) of their treatment, to avoid the potential distress or motor complications associated with an interruption of their medication and because of logistic constraints. No dyskinesia was observed (during MDS-UPDRS III motor examination) at the time of the visit. Local ethics committee approved this study (IRB Paris VI, RCB: 2014-A00725-42) and all participants gave written informed consent.

A total of 148 participants (one session per subject) were kept for the analyses, comprising 104 PD (68M, 36F) and 44 HC (23M, 21F). Among them, 146 underwent rs-fMRI (103 PD patients [67M, 36F] and 43 HC [22M, 21F]), and 78 completed a DaT-SPECT (48 PD [28M, 20F] and 30 HC [13M, 17F]).

### Clinical examination

Clinical examination included the Hoehn and Yahr scale^61^, the Movement Disorder Society Unified Parkinson’s Disease Rating Scale (MDS-UPDRS; parts I, II, III in the OFF and ON states, and IV)^62^, the Mattis dementia rating scale (DRS)^63^, and the Montreal Cognitive Assessment score (MoCA)^64^. All subjects’ clinical scores were measured on the same day as the voice recordings.

### Speech data

All participants were recorded in consultation rooms in the clinical investigation center at the Paris Brain Institute (ICM), or in the Pitié-Salpêtrière Hospital sleep disorder unit (Paris, France). The microphone was a professional head-mounted omnidirectional condenser microphone (Beyerdynamic Opus 55 Mk II) located approximately 10 cm from the subject’s mouth and connected to a professional sound card (Scarlett 2i2, Focusrite). Speech data were sampled at a rate of 96,000 Hz with 24-bit resolution, covering a frequency bandwidth of 50–20,000 Hz. Speech recordings were obtained during a single 20-min session per subject, with the assistance of a medical experimenter. The speech tasks were presented in a randomized order via a graphical user interface and included vocalizing sustained vowels, spontaneous speech, rapid and slow syllable repetitions at a specified pace, short sentence repetitions, and reading tasks^5^.

Different quantitative acoustic features were automatically extracted from the speech recordings using PRAAT^65^, MATLAB (*The MathWorks, Inc., Natick, MA,USA*), and Kaldi^66^ software. These features were related to voice, speech, and language production, including prosody, verbal fluency, rhythm-following ability, phonation, and articulation. Pre-processing steps involving spectral subtraction^67^ were conducted before feature extraction to eliminate additive and stationary background noise. To quantify prosodic impairments, pitch variations (melody) and intensity variations (stress on words or syllables) were analyzed. Pitch variations were measured by the standard deviation of the base-10 logarithm of fundamental frequency *(SD log F0)*, calculated across reading, sentence repetition, and free speech tasks. Intensity fluctuations were evaluated by the standard deviation of the sound intensity level *(SD Int)* during dialog reading. Mean intensity was excluded from the analysis because of methodological constraints (microphone placement variability, probable drift in microphone sensitivity over time) and environmental factors (hospital ambient noise), which prevented a reliable and interpretable measure. To examine verbal fluency disruptions, the median pause duration (*Pause duration*) during the free speech task was calculated. To evaluate rhythmic abilities, participants were instructed to repeat specific syllables (/pa/ and /ku/) for 30 s at a steady pace of one syllable per second, following a 5-s example provided beforehand. The ability to maintain a steady rhythm was analyzed as the relative standard deviation of pace (*RSD rhythm*), and the ability to globally keep an imposed pace was derived from the difference between the average pace and the given pace (*Absolute rhythm*). To assess phonation impairments, three types of voice measures were extracted using the sustained vowel /a/ tasks: jitter, shimmer, and Noise-to-Harmonics Ratio. These features characterized pitch instability, intensity instability, and voice harmony, respectively. Due to the inherent correlation among jitter, shimmer, and the Noise-to-Harmonics Ratio, a composite phonation feature (Phonation score) was constructed by averaging the standardized values of these features. Further details about the speech features and the signal processing algorithms used for their calculation are provided in a previous article^5^. Finally, to quantify articulatory impairments, we used the scores resulting from classifiers based on the MFCCs, which characterize the spectral envelope (*Articulation score*). We chose appropriate classification techniques depending on the sex, namely an MFCC-Gaussian Mixture Model for males^68^ and a classifier using x-vectors for females^7^. The speech features used in this study are summarized and described in Table 7.

**Table 7:** Description of speech features

Speech features	Speech tasks	Description
*Prosody*		
SD log_10_ F0 (x10^−3^)	Free speech Sentence repetitions Readings	Standard deviation of the base-10 logarithm of fundamental frequency (pitch variations)
SD Int (dB)	Dialog reading	Standard deviation of intensity (intensity variations)
*Pauses*		
Median duration (s)	Free speech	Median duration of the pauses >200 ms
*Rhythm*		
RSD	Slow syllable repetitions	Relative standard deviation of pace
Absolute (s)	Slow syllable repetitions	Difference between the average pace and the given pace
*Phonation*		
Phonation score	Sustained vowel	Composite score of standardized NHR, jitter (short-term pitch fluctuations), and shimmer (short-term intensity fluctuations)
*Articulation*		
Articulation score	Sentence repetitions Readings Fast syllable repetitions	PD vs HC classification score based on Mel-Frequency Cepstral Coefficients (MFCCs)

*F0* fundamental frequency, *Int* Intensity, *NHR* Noise-to-Harmonics Ratio, *RSD* relative standard deviation, *SD* standard deviation.

### Neuroimaging

Participants underwent MRI on a Siemens Prisma 3 T system (Siemens Healthcare, Erlangen, Germany) using a 64-channel receive-only head coil. The following images were acquired: whole-brain T1-weighted three-dimensional (3D) magnetization-prepared two rapid gradient echo (MP2RAGE; 1-mm isotropic voxel size), and resting state blood oxygen level-dependent (BOLD) echo planar images (EPI) (44 oblique axial slices, repetition time/echo time/flip angle = 2.19 s/25 ms/90°, voxel size = 2.8 × 2.8 × 2.8 mm^3^, 220 volumes). During rs-fMRI, the participants kept their eyes closed and had to relax while remaining awake. A field map was acquired to correct for the EPI geometric distortion. More details about the parameter settings can be found in Table S1.

DaT-SPECT imaging (hybrid gamma camera Discovery 670 Pro system; GE Healthcare, Chicago, IL) was performed using the ^123^I-FP-CIT tracer (DaTScan^TM^; 185-200 MBq) (circular orbit, clockwise rotation, zoom factor = 1.5/1.5, matrix size = 128 × 128 voxels, number of slices = 128, voxel size = 2.9 × 2.9 mm^2^, slice thickness = 2.9 mm, rotations = 1, number of frames in rotation = 120, actual frame duration = 30 s, energy window = 143.1–174.9 keV). Participants received thyroid gland blockers via one dose of Lugol regimen and an intravenous injection of ^123^I-FP-CIT tracer, followed by a SPECT scan performed between 3 and 4 h after the tracer injection.

When not otherwise stated, all pre-processing and analyses were carried out using MATLAB (R2017b, The MathWorks, Natick, MA, USA).

DaT-SPECT images were reconstructed on the GE Healthcare Xeleris Workstation using an iterative algorithm with spatial filtering (fourth-order low-pass filter with a cut-off frequency equal to 3.5 mm^−1^) and Chang attenuation correction (*μ* = 0.12 cm^−1^)^69^. The DaT-SPECT images were rigidly aligned to the corresponding T1-weighted anatomical MRI via the corresponding computed tomography image. All image coregistrations were performed using NiftyReg^70,71^.

Resting state fMRI data were preprocessed using Statistical Parametric Mapping (SPM12; http://www.fil.ion.ucl.ac.uk/spm/) with spatial realignment for head motion correction, slice timing, spatial normalization, and smoothing. Nuisance regressors were individually defined using the PhysIO toolbox^72^ in the Translational Algorithms for Psychiatry-Advancing Science (TAPAS) software package^73^. This toolbox is based on Framewise Displacement (FD) that evaluates abrupt volume-to-volume displacements >0.5 mm, which was inputted into the first-level analyses. The nuisance regressors included the white matter signal, cerebrospinal fluid signal (CSF), motion parameters with their derivatives (24 regressors), and stick functions to exclude volumes with FD > 0.5 mm. Individual anatomical and realigned functional images were normalized to the Montreal Neurological Institute (MNI) space using DARTEL and smoothed with an 8-mm Gaussian filter. Temporal bandpass (0.01–0.1 Hz) filtering was then used to remove low-frequency drifts and physiological high-frequency noise.

Our analyses were focused on the striatum and its relationship to cortical language networks using a region of interest (ROI)-based approach. For striatal regions, the sensorimotor and associative territories of the left and right putamen and caudate nucleus were automatically segmented by aligning a digital template of the human basal ganglia (YeB atlas)^74^ to the anatomical T1-weighted MRI (Supplementary Fig. S1). For cortical regions, two masks were defined: the first included bilateral brain areas belonging to the sensorimotor language network, and the second included bilateral brain areas belonging to the associative language network. Both these networks were defined based on the language-specialized SENtence Supramodal Areas AtlaS (SENSAAS atlas)^75^, the Atlas of Intrinsic Connectivity of Homotopic Areas (AICHA atlas)^76^, and Automated Anatomical Labelling Atlas 3 (AAL3) for the thalamic relays. The associative mask was composed of the following bilateral regions: the pre-SMA, the inferior frontal sulcus, the superior and inferior frontal gyri (including the pars orbitalis and pars triangularis), the precentral gyrus, the supramarginal gyrus, the angular gyrus, the anterior insular gyrus, the superior and middle temporal gyri, and the ventral anterior thalamic nucleus (Fig. S2). The sensorimotor mask was composed of the following bilateral regions: the precentral gyrus, the SMA, the posterior putamen, and the ventrolateral thalamic nucleus (Fig. S3).

For each subject, the DaT-SPECT image was processed according to a previously detailed pipeline^77,78^. Briefly, after identification of the striatal ROIs, the following steps were performed: correction for partial volume artifacts, quantitative DaT referencing relative to a region in the occipital cortex with non-specific tracer uptake, and regional calculation of the average striatal binding ratio (SBR).

A seed-based connectivity analysis was performed using SPM12 (first-level analysis) between the eight striatal seeds (two territories: associative, sensorimotor; two striatal regions: caudate, putamen; two hemispheres: left, right) and the corresponding (associative or sensorimotor) language masks. For each participant, general linear modeling of the BOLD signal of each voxel within the language masks was performed using the time course of the corresponding striatal seed (averaged over the voxels in the seed mask) as a regressor, alongside nuisance covariates. For each subject and each striatal seed, a functional connectivity map was obtained. These individual functional connectivity maps, hereafter referred to as “striatal maps,” were transformed into individual z-score maps and included as inputs in the group analysis.

### Statistical analysis

Prior to conducting the statistical analyses, the normality of the data distribution was assessed (Shapiro-Wilk test). Based on these results, parametric or non-parametric tests were applied accordingly.

Group analyses were conducted using univariate two-way analyses of variance (ANOVAs), with disease status and sex as factors, and including the interaction between both in the model. As age and education level did not show any significant difference between PD and HC, they were not included as nuisance parameters for the group analyses. For speech analyses, ANOVAs were performed for each speech feature. Regarding DaT-SPECT, ANOVAs were computed from the SBR of the eight previously defined anatomical and functional regions: associative and sensorimotor putamen (left and right) and associative and sensorimotor caudate nucleus (left and right). *P* values were adjusted with the FDR method to account for multiple comparisons. Differences were considered significant at a corrected p < 0.05. For rs-fMRI statistical analysis, ANOVAs were defined within the SPM12 framework, incorporating the individual striatal maps as input in the model (second-level analysis). Functional connectivity group differences were considered significant at the threshold of *p* < 0.001 uncorrected at the voxel-based level and *p* < 0.05 with family-wise error (FWE) correction at the cluster level. Effect sizes for each significant effect were estimated using partial eta-squared (*η*²), derived from the corresponding *F*-statistics (from DaT-SPECT regional analyses or at the peak voxel of significant fMRI clusters) and their associated degrees of freedom. For each *η*², a 95% confidence interval was computed using the noncentral F distribution.

Correlation analyses were performed in PD patients, between the discriminant speech features (according to the two-way ANOVA) and neuroimaging data. Males and females were analyzed separately to examine the neural correlates, potentially sex-dependent, of PD-related speech impairments.

For *DaT-SBR*, partial Pearson correlations were calculated considering the individual DaT-SPECT SBR values of the four striatal regions (two territories: associative, sensorimotor; two striatal regions: caudate, putamen). The correlations were controlled for age, as it may affect both speech^79^ and SBR values^80^. Control was also applied for the time delay between the voice recording and DaT-SPECT to prevent an additional source of unexplained variability. *P* values were FDR adjusted to compensate for the multiple comparisons. Differences were considered significant at corrected *p* < 0.05.

Regarding *functional connectivity*, multiple regression analyses were conducted for each striatal seed region, with the discriminant speech features as predictors and the individual functional connectivity maps as dependent variables. Both age and education level were included as covariates of no interest to control for their effects, as they may influence both speech^81^ and the functional connectivity^82^ in the corresponding neural networks. For the same reasons as with the DaT-SPECT, the correlations were also controlled for the time delay between the voice recording and the fMRI scan. Correlations were considered significant at the threshold of *p* < 0.001 uncorrected at the voxel level and *p* < 0.05 with FWE correction at the cluster level.

Effect sizes for each significant effect were estimated using the partial correlation coefficient (*r*), derived from the corresponding t-statistics (from DaT-SPECT regional analyses or at the peak voxel of significant fMRI clusters) and their associated degrees of freedom. For each *r*, a 95% confidence interval was computed using Fisher’s *z* transformation.

We did not include the Levodopa Equivalent Daily Dose (LEDD) as a covariate in our main analyses because of its collinearity with disease severity. By controlling for LEDD, we would risk removing a significant portion of the variance directly related to the disease progression and its underlying pathology.

## Supplementary information

Supplementary Materials

## Data Availability

The datasets presented in this article are not publicly available because of restrictions under the participants' informed consent. Requests to access the datasets should be directed to M.V. [marie.vidailhet@aphp.fr].
