# Supplementary material for: Striatal network dysfunction underlying speech impairment in early Parkinson’s disease
Source: NPJ Parkinsons Dis. 2026 Oct 12;12:251. doi: 10.1038/s41531-026-01509-2 (PMC13669323; doi:10.1038/s41531-026-01509-2)
Supplement: Supplementary file 1 [file 41531_2026_1509_MOESM1_ESM.pdf]

SUPPLEMENTARY TABLES

Table S1. Magnetic resonance imaging protocols

	T1-W anatomical MRI	T1-W neuromelanin MRI	Rs-fMRI
**Protocol**	3D MP2RAGE	2D TSE	2D BOLD-EPI
**Slice orientation**	Sagittal	Axial transverse, perpendicular to the longitudinal axis of the brainstem	Axial transverse
**Scan mode**	3D	2D	2D
**Phase encoding direction**	Anterior-posterior	Anterior-posterior	Anterior-posterior
**Repetition time (ms)**	5000	890	2190
**Echo time (ms)**	2.98	13	25
**Echo train lengh**	n/a	3	37
**Inversion time (ms)**	700 and 2500	n/a	n/a
**Flip angle (degrees)**	4 and 5	90 and 180 (refocus)	90
**Number of excitations**	1	3	1
**FoV:** anterior - posterior x right - left x superior - inferior (mm^3)	256 x 231 x 176	200 x 220 x 45	213 x 213 x 123
**Bandwith (Hz/pixel)**	240	160	2435
**Acquisition matrix**	256 x 232	512 x 464	76 x 76
**Slice thickness (mm)**	1	3	3
**Number of volumes**	1	1	220
**Number of slices**	255	15	44
**Acquired voxel size (mm)**	1x1x1	0.43 x 0.43 x 3	2.8 x 2.8 x 3
**Acquisition time (min:s)**	08:12	06:55	08:08

Parameter settings for all the MRI sequences
BOLD = Blood oxygenation level dependent imaging; FoV = field of view; MP2RAGE = magnetization prepared two rapid acquisition gradient echoes; n/a = not applicable; Rs-fMRI: resting state functional MRI; TE = echo time; TI = inversion time; TR = repetition time; TSE = turbo spin echo

SUPPLEMENTARY FIGURES

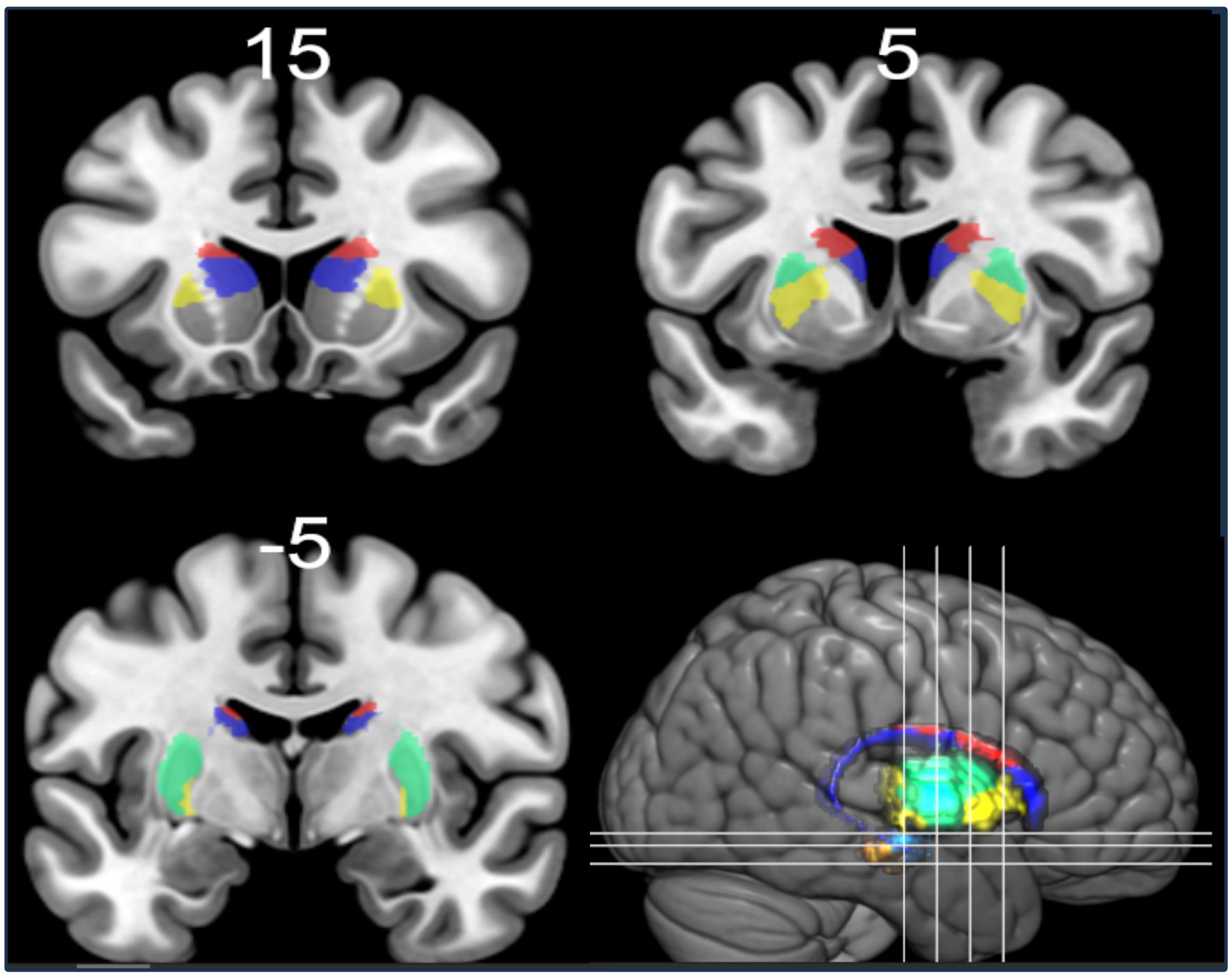

Figure S1: Basal ganglia seed definitions. Masks for the associative and sensorimotor subregion of basal ganglia were derived from the YEB atlas for the putamen and the caudate. Basal ganglia template regions overlaid onto the MNI template brain. The numbers correspond to the MNI Y-coordinates (coronal views).
Color legend: associative caudate represented in red, sensorimotor caudate represented in blue, associative putamen represented in green and sensorimotor putamen represented in yellow.

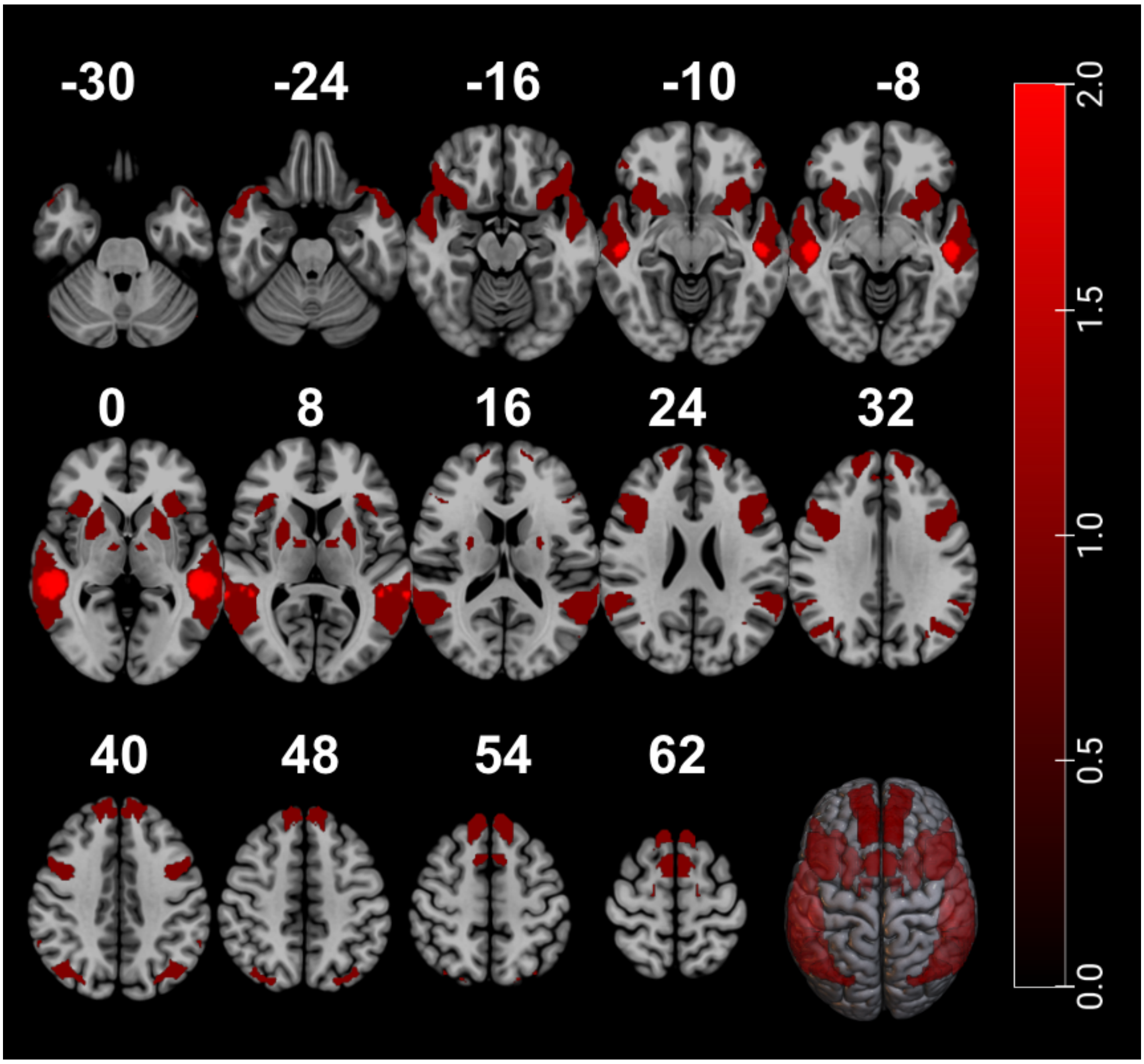

Figure S2: Associative language network templates overlaid onto the MNI template brain.

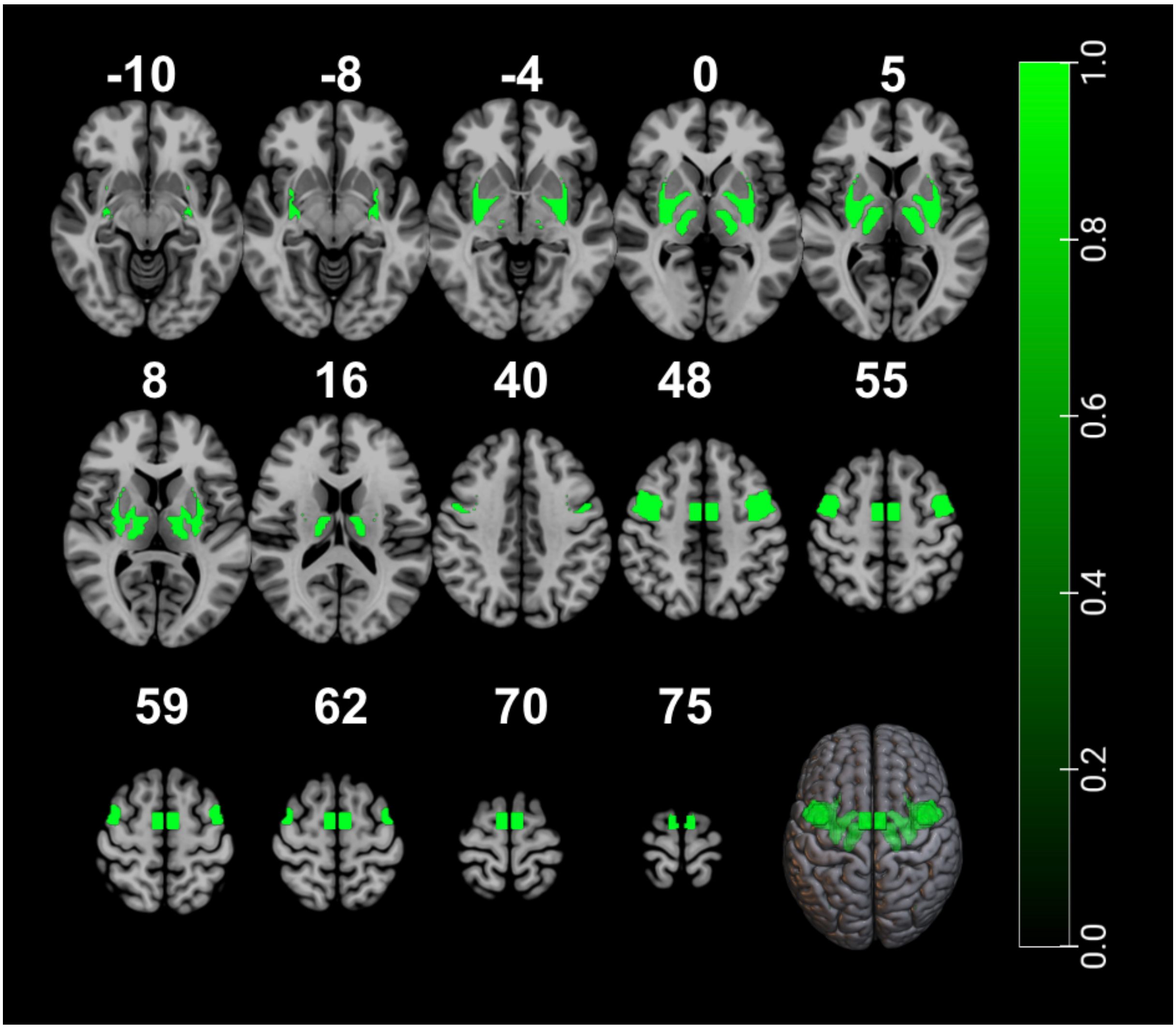

Figure S3: Sensorimotor language network templates overlaid onto the MNI template brain.
